# Digital Remote Monitoring Using an mHealth Solution for Survivors of Cancer: Protocol for a Pilot Observational Study

**DOI:** 10.2196/52957

**Published:** 2024-04-30

**Authors:** Pasquale F Innominato, Jamie H Macdonald, Wendy Saxton, Laura Longshaw, Rachel Granger, Iman Naja, Carlo Allocca, Ruth Edwards, Solah Rasheed, Frans Folkvord, Jordi de Batlle, Rohit Ail, Enrico Motta, Catherine Bale, Claire Fuller, Anna P Mullard, Christian P Subbe, Dawn Griffiths, Nicholas I Wreglesworth, Leandro Pecchia, Giuseppe Fico, Alessio Antonini

**Affiliations:** 1 Oncology Department Ysbyty Gwynedd Betsi Cadwaladr University Health Board Bangor United Kingdom; 2 Warwick Medical School & Cancer Research Centre University of Warwick Coventry United Kingdom; 3 Chronotherapy, Cancers and Transplantation Research Unit, Faculty of Medicine Université Paris-Saclay Villejuif France; 4 Institute for Applied Human Physiology, School of Psychology and Sports Science Bangor University Bangor United Kingdom; 5 Research and Development Department Ysbyty Gwynedd Betsi Cadwaladr University Health Board Bangor United Kingdom; 6 Knowledge Media Institute The Open University Milton Keynes United Kingdom; 7 Health Innovation Samsung Staines United Kingdom; 8 Dietetics Department Ysbyty Gwynedd Betsi Cadwaladr University Health Board Bangor United Kingdom; 9 PredictBy Barcelona Spain; 10 Tilburg School of Humanities and Digital Sciences Tilburg University Tilburg Netherlands; 11 Institut de Recerca Biomèdica de Lleida Lleida Spain; 12 Acute and Critical Care Medicine Ysbyty Gwynedd Betsi Cadwaladr University Health Board Bangor United Kingdom; 13 School of Medical Sciences Bangor University Bangor United Kingdom; 14 School of Engineering University of Warwick Coventry United Kingdom; 15 Facoltà Dipartimentale di Ingegneria Università Campus Bio-Medico di Roma Rome Italy; 16 Life Supporting Technologies, Escuela Técnica Superior de Ingenieros de Telecomunicaciones Universidad Politécnica de Madrid Madrid Spain

**Keywords:** cancer, survivorship, artificial intelligence, remote monitoring, mobile health, mHealth, digital health, circadian, actigraphy, mobile phone

## Abstract

**Background:**

Healthy lifestyle interventions have a positive impact on multiple disease trajectories, including cancer-related outcomes. Specifically, appropriate habitual physical activity, adequate sleep, and a regular wholesome diet are of paramount importance for the wellness and supportive care of survivors of cancer. Mobile health (mHealth) apps have the potential to support novel tailored lifestyle interventions.

**Objective:**

This observational pilot study aims to assess the feasibility of mHealth multidimensional longitudinal monitoring in survivors of cancer. The primary objective is to test the compliance (user engagement) with the monitoring solution. Secondary objectives include recording clinically relevant subjective and objective measures collected through the digital solution.

**Methods:**

This is a monocentric pilot study taking place in Bangor, Wales, United Kingdom. We plan to enroll up to 100 adult survivors of cancer not receiving toxic anticancer treatment, who will provide self-reported behavioral data recorded via a dedicated app and validated questionnaires and objective data automatically collected by a paired smartwatch over 16 weeks. The participants will continue with their normal routine surveillance care for their cancer. The primary end point is feasibility (eg, mHealth monitoring acceptability). Composite secondary end points include clinically relevant patient-reported outcome measures (eg, the Edmonton Symptom Assessment System score) and objective physiological measures (eg, step counts). This trial received a favorable ethical review in May 2023 (Integrated Research Application System 301068).

**Results:**

This study is part of an array of pilots within a European Union funded project, entitled “GATEKEEPER,” conducted at different sites across Europe and covering various chronic diseases. Study accrual is anticipated to commence in January 2024 and continue until June 2024. It is hypothesized that mHealth monitoring will be feasible in survivors of cancer; specifically, at least 50% (50/100) of the participants will engage with the app at least once a week in 8 of the 16 study weeks.

**Conclusions:**

In a population with potentially complex clinical needs, this pilot study will test the feasibility of multidimensional remote monitoring of patient-reported outcomes and physiological parameters. Satisfactory compliance with the use of the app and smartwatch, whether confirmed or infirmed through this study, will be propaedeutic to the development of innovative mHealth interventions in survivors of cancer.

**International Registered Report Identifier (IRRID):**

PRR1-10.2196/52957

## Introduction

### Background

Cancer survivorship begins with the diagnosis and continues throughout the whole life span of the person [1]. Owing to repeated advances in anticancer and supportive treatments, survivors of cancer represent an ever-growing population [2]. Indeed, accelerated by the recent COVID-19 pandemic, alternative models of care for survivors of cancer are being proposed to meet the growing demands, driven by patient-specific issues and local health care resource constraints [3]. Survivors of cancer could be still receiving anticancer treatment, or they may have already completed treatment, albeit only temporarily. In the former case, acute toxicities from anticancer treatment might be the most relevant and bothersome, whereas in the latter case, delayed and long-lasting toxicities and a plethora of physical, emotional, social, practical, and informational issues become the most critical unmet care needs for their well-being [4].

Healthy lifestyle interventions have a positive impact on cancer-related outcomes, such as overall survival, tolerance to treatment, and health-related quality of life (HRQoL), as well as other metabolic and degenerative diseases [5]. Specifically, appropriate habitual physical activity, adequate sleep, and a regular wholesome diet are of paramount importance for the wellness of survivors of cancer [1] and are considered fundamental pillars of supportive care in survivors of cancer [6]. Thus, the promotion of interventions for healthier habitual nutritional, sleep, and physical activity behaviors is broadly recommended [7]. Nevertheless, there are motivational, practical, and informational barriers to the wide adoption of modifiable healthy lifestyle behaviors in survivors of cancer [8-10].

As a class of ubiquitous technologies, mobile health (mHealth) apps have the potential to support lifestyle interventions. The combination of wearables and smartphone sensors allows scalable and practically implementable study of human behavior and physiology [11]. Automatic collection of personal health data can be complemented by self-reported assessments delivered through mHealth apps, allowing real-time monitoring of patient-reported outcomes. Such a combination of mHealth data with electronic medical records and electronic health records also opens the possibility of a new breed of artificial intelligence (AI) apps, with the potential to facilitate early detection of new conditions, identify worsening of symptoms, and provide novel delivery methods for lifestyle interventions.

### Objectives

The European Union’s (EU’s) Horizon 2020 GATEKEEPER (GK) project is a flagship innovation action aimed at implementing this vision and delivering the future EU data and AI infrastructure for health care solutions [12]. GK has several goals, ranging from the technical software architecture and cross-countries’ and cross-organizations’ information security and data policy to the evaluation of data-driven services and AI-based interventions. GK’s key innovation is combining health medical records with medical sensors and non–medical data from mHealth apps, wearables, and smart devices. The intuition is that apps and devices would bring critical contextual and behavioral information that, combined with medical data and knowledge, would lead to a breakthrough in AI for health care. For instance, ongoing investigations within the GK project involve the development of risk prediction algorithms for mental health and cognitive impairment and digital coaching to mitigate loneliness-related risks [13]. These breakthroughs are the result of this new approach and the data sets collected as part of the project.

In this regard, GK includes a multicentric large-scale pilot across 8 EU and 3 Asian countries for a total of 11 Reference Pilot Sites. The large-scale pilot aims to develop and test a novel approach to the evaluation of digital innovation in health care. The proposed approach can be described as an integrative analysis of cost-benefits, combining the local evaluation of the wide range of approaches and solutions at the reference sites with 10 main health care themes [13]. Pilots within the GK project evaluate, monitor, or treat the most prevalent, chronic, noncommunicable conditions according to the World Health Organization, including cardiovascular, metabolic, and respiratory diseases, whereas this pilot study focuses on cancer [14].

Specifically, the proposed study is 1 of the 3 UK Reference Pilot Sites and falls under the Reference Use Cases on *co-morbidity and polymedication*. In preparation for the proposed study, a new mHealth solution for survivors of cancer was co-designed. This solution was initially tested in a small-scale study focused on the use of a wearable mHealth app before further expansion within the context of GK [13].

In the proposed study, behavioral data (nutritional intake, physical activity, and sleep) will be obtained through self-reporting (recorded via an app) and automatic collection (by a wearable smartwatch). The data will then be used for remote monitoring of cancer symptoms, automatic assessments of biometric data, and integration with health medical records. Thus, the aim of this study is to assess the feasibility of mHealth monitoring in survivors of cancer. It is hypothesized that mHealth monitoring will be feasible in survivors of cancer; specifically, at least 50% (50/100) of the participants will engage with the GK app at least once a week in 8 of the 16 study weeks.

## Methods

### Ethical Considerations

The study protocol has been approved by the National Health Services (NHS) Research Ethics Committee (Integrated Research Application System ID: 301068; Research Ethics Committee reference: 22//SC/0323) and therefore by the Health Research Authority and Health and Care Research Wales.

Informed consent describes both the primary and secondary uses of data. Concerning secondary uses, participants are informed about the full anonymization of their data with the aim of creating a resource for the study of predictors of symptom worsening, cancer relapse, and hospitalization events. The informed consent form is provided both in English and Welsh.

The study approval included a comprehensive data protection impact assessment and a data management plan concerning data flow, processing, and sharing. In summary, participants will be given a study code and an anonymous user account for the use of the wearable device and study app. The study will only use biometric data generated through commercially available systems—Samsung Health (SH) app and Samsung Galaxy Watch. The mHealth solution decouples and anonymizes data from their data vault on patients’ phones before transfer and processing. Only the hospital staff will have access to the identity or personal information of patients, and no other researchers involved will be able to access the registry of patient identifiers.

Participants will be gifted with the wearable device (smartwatch) and replacement Android phone (provided to iOS users). Furthermore, a form of compensation is the training in the use of these widely available technologies as well as the opportunity to learn more about how digital coaching and digital health technologies make use of personal data. Finally, the study will provide participants with the opportunity to reflect on the role of self-management and potential support available to them.

### Study Setting

This study is the result of a co-design approach involving the Betsi Cadwaldr University Health Board Cancer Services; specialists on nutrition, sleep, and physical activity; technology providers; AI researchers; experts on clinical research and evaluation; acute medicine physicians; and patients with cancer. Over a period of 2 years, weekly meetings between main investigators and technical partners, focus groups with patients with cancer, and a cocreation group involving all stakeholders generated a patient-facing app, a clinician facing dashboard, AI algorithms, communication and training materials, and specialist outcome assessments, which led to an mHealth solution that could be pragmatically implemented alongside current routine care for survivors of cancer, as provided by the public health care system, NHS Wales.

The study design is also the result of learning from other GK Reference Pilot Sites. In this regard, we propose a feasibility design with a duration of 16 weeks, which will be conducted at a single institution, the Oncology Service of Ysbyty Gwynedd Hospital, Bangor, Wales, United Kingdom.

### Eligibility Criteria

To be enrolled in the study, all participants must meet all the following requirements: (1) diagnosis of any solid malignancy, at any stage; (2) completion of treatment (including surgery, radiotherapy, and any type of systemic anticancer treatment); (3) clinical, biochemical, and radiological confirmation of controlled disease (no evidence of residual disease or nonprogressive disease); (4) scheduled for either a therapeutic break (from systemic anticancer treatment) or having completed the planned multimodal treatment; (5) scheduled for active surveillance plan within the oncology service; (6) male or female patients aged at least 18 years; and (7) scored World Health Organization performance status 0 to 2 [15].

Patients will be excluded from participating in the study if they meet any of the following criteria: (1) not possessing a SIM card, (2) no internet access through Wi-Fi or unwilling to use mobile data allowance on a daily basis, (3) inability to understand and follow the instructions autonomously, (4) uncontrolled severe comorbidities (eg, cardiovascular, metabolic, neurological, and respiratory), (5) lack of capacity, (6) inability to understand English, (7) still undergoing systemic anticancer treatment (apart from bone-targeted agents, hormonal treatment against breast or prostate cancers, or maintenance monoclonal antibody monotherapy), (8) poor general condition (performance status>2), and (9) inability to sign informed consent.

We decided to focus on survivors of cancer who were not receiving anticancer treatment to minimize the potential impact of treatment-induced fatigue or anorexia. On the basis of previous discussion with patients with cancer, it was felt that this population was the most suitable to test our mHealth solution in the first place, which can be adapted in future studies to the needs of patients on active disease-modifying treatment.

### Digital Health Care Technology

#### SH App and Samsung Galaxy Watch

The SH app, coupled with Samsung Galaxy Watch, can track a number of parameters, including (1) food and water intake (including calorie tracking), (2) sleep patterns, (3) blood oxygen saturation levels, (4) body composition, (5) body weight, (6) calorie intake, (7) heart rate, (8) blood pressure, and (9) habitual physical activity (step counts and minutes of moderate and physical activity completed per day).

The SH app uses biometrics obtained by the connected wearable device, the smartphone itself, and self-reporting to record variables such as accelerometry, heart rate, time, distance traveled, and speed. Data are then used for activity tracking by matching the signature characteristics of a wide range of exercises (such as walking, hiking, or swimming) to calculate step counts, the number of minutes of moderate and vigorous physical activity completed, and the number of calories expended. The SH app supports tracking of food intake during the day. For instance, users can record meals and snacks as well as fluid and caffeine intake. Macronutrients (proteins, carbohydrates, and fats) and micronutrients (vitamins and minerals) can be calculated. The SH app also implements sleep analysis, sleep duration tracking, and sleep stages to provide insights about the quantity and quality of sleep.

#### GK App

The GK app is an mHealth solution that will be installed on a personal smartphone and paired with the SH app. Patients will use it to periodically fill self-assessment questionnaires and view data visualization analytics.

#### Web Portals and Tablets

To complement the data collected by the SH app, Samsung Galaxy watch, and GK app, the patients will fill in questionnaires. These questionnaires will be completed by patients using a tablet, and paper forms will be provided in cases where a tablet is not available. The questionnaires will cover data describing HRQoL, symptoms, sleep, and patient feedback on care. Patients will also use an web-based survey platform (Qualtrics) to fill in questionnaires about habitual physical activity, any structured exercise completed, and patient activation.

### Recruitment Program

Participants will be identified from oncology services at Ysbyty Gwynedd Hospital, Betsi Cadwaladr University Health Board, by reviewing the outpatient clinic lists of each participating oncologist, advanced nurse practitioner, and clinical pharmacist on a daily basis. For potentially eligible patients, the appropriateness of proposing participation in this trial will be decided by the lead oncologist. At the end of the clinical consultation, if deemed appropriate, the clinician will introduce the study and provide the information leaflet, and a date in the future will be set for the volunteer to meet a member of the clinical research team to answer all the possible queries and to confirm sign-up for participation. If and when a participant agrees to proceed with the study, they will be asked to sign the consent form and will receive the smartwatch (and a smartphone, if required). The clinical research team member will help with the setup of the apps and smartwatch pairing and will provide a technical overview of both as well as the troubleshooting and contact information. Baseline questionnaires will be completed on a dedicated tablet or paper as per the recommended procedures with a standard approach to collect these patient-generated data with the supervision of the clinical research nurse or officer [16]. The same approach will be used to collect end-of-study questionnaires data. The expected recruitment period is January 2024 to June 2024, with the maximum duration of data collection for each participant set to up to 16 weeks. We anticipate being able to recruit about 5 patients per week over a 24-week period to reach our target.

### Contingency Plans for Potential Recruitment Risks

For this monocentric pilot, we have built on previous experience within the oncology department with digital monitoring studies [17-19], and the estimated recruitment has been discussed at length between clinicians and research team members. A risk assessment analysis has been performed, and no transfer, avoidance, or prevention of the risk of poor recruitment has been identified. To mitigate the risk of lower than expected acceptance from the patients, we have trained the involved clinician to present the study to their patients, who were potentially eligible for participation, rather than simply referring them to the research team for study explanation. To mitigate the risk of poor engagement from the clinicians themselves, we have already involved them since the beginning of the study, and we have planned meetings between the delivery research team and clinicians after multidisciplinary team meetings to remind them of the study objectives and inclusion criteria. We have also printed a leaflet containing the main study features for each relevant outpatient clinic. Finally, we decided to accept the risk of slow recruitment as, within the GK project, we have no further studies pending upon the results of this study.

### Study Procedures

Following the provision of written informed consent, the following measures will be obtained and recorded in the case report form (CRFs) at baseline and at the end of the study (16 weeks):

- Height (only measured at study entry)
- Body mass
- BMI
- Waist circumference
- Hip circumference
- Medical history
- Concurrent conditions
- Concomitant medications
- WHO performance status [15]
- Cancer type and stage
- Harms—adverse events.

We will record and report “unexpected” serious adverse events resulting in death, life-threatening complications, hospitalization or prolongation of existing hospitalization, persistent or significant disability or incapacity, congenital abnormality or birth defect, and other “important medical events” that are considered serious if they jeopardize the participant or require an intervention to prevent 1 of the abovementioned consequences.

The following adverse events are considered to be “expected”: anxiety related to the monitoring of symptoms, diet, and exercise; distress related to the regular use of digital well-being technology; cutaneous allergic reactions to the smartwatch; and visual strain with the light-emitting smartphone screen.

The study uses the following questionnaires:

- EQ-5D-5L: Participants’ HRQoL will be measured using the EQ-5D-5L questionnaire [20]. EQ-5D-5L is a widely used tool for assessing the domains of mobility, self-care, usual activities, pain or discomfort, and anxiety or depression as well as recording patients’ self-rated health on a visual analog scale.
- Multidimensional Fatigue Inventory Short Form: Multidimensional Fatigue Inventory Short Form [21] will be used to assess fatigue. This is a validated short form of the more in-depth Multidimensional Fatigue Inventory questionnaire [22] and uses 10 simple questions to assess physical, emotional, and cognitive fatigue [21].
- Satisfaction, Alertness, Timing, Efficiency and Duration (SATED): The SATED questionnaire [23] will be used to assess subjective sleep. SATED is a simple questionnaire to determine the degree of sleep fulfillment and to measure the dimensions of sleep health based on 5 simple questions [24]. It has been used in >150 peer-reviewed papers and studies.
- Patient Assessment of Chronic Illness Care: Patient Assessment of Chronic Illness Care questionnaire [25] will be used to assess experience with care. It is recommended for research and quality improvement and has been successfully deployed in digital oncology studies [26].
- Edmonton Symptom Assessment System (ESAS; digital form completed daily through the GK app): The global symptom burden will be assessed through ESAS [27]. ESAS has a recall time of 24 hours and is adapted to daily completion [28]. Daily total symptom distress scores (ESAS physical score+emotional score+well-being) [29] will be computed, and their temporal trajectories will be compared using general linear and random mixed effects models for longitudinal data [30].
- Global Physical Activity Questionnaire: Habitual physical activity will be assessed using the Global Physical Activity Questionnaire short form [31] and four unvalidated questions: (1) minutes of moderate physical activity completed per week, (2) minutes of vigorous physical activity completed per week, (3) number of sessions of strength training completed per week, and (4) number of sessions of balance training completed per week.
- Patient Activation Measure–13: Patient activation, an individual’s knowledge, skills, and confidence integral to managing their own health and health care, will be assessed using the Patient Activation Measure–13 [32].

Table 1 lists the timeline and type of data collected throughout the study period.

**Figure 1:**
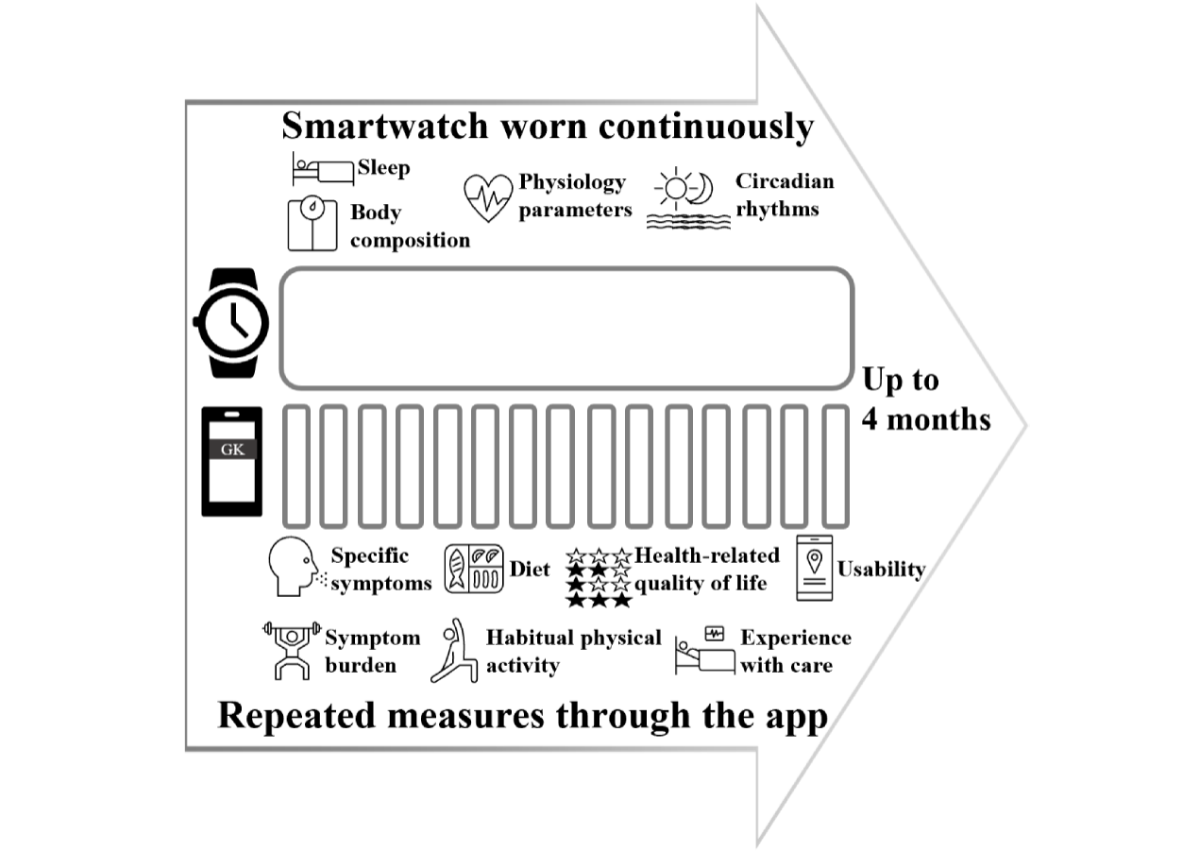
Study schematic of the trial design (longitudinal and observational) with features of the patient-generated data collected through the smartwatch (objective and mostly continuous) and app (subjective and repeated measures or snapshots).

Participants will then be presented with the mHealth solution: the GK app on a personal smartphone and a smartwatch paired with the SH app. The participant will be trained to use the apps and watch. The GK app features a set of interactive questionnaires to collect the baseline and periodic self-assessment data. Furthermore, the GK app provides timely reminders about the need to complete the self-assessments and data visualization analytics. Finally, the GK app collects and anonymizes data from the smartwatch and SH app for daily data collection on a dedicated remote server. The smartwatch adopted can be configured and used in different ways; each patient will be able to set up complementary apps offering extra functionalities such as the stress level analysis and to identify the preferred patterns of use. Overall, the data generated by the smartwatch will be collected and aggregated on a daily basis, including heart rate, sleep and physical activity patterns, and calories and food intake. The sampling of data will be dependent on the user settings and contingencies, such as charging at night or battery level. Throughout the 16-week study period, the smartwatch will continuously and unobtrusively record habitual physical activity, sleep patterns, energy expenditure, step counts, and oxygen saturation through its biosensors (Figure 1). For example, the amount of moderate and vigorous physical activity completed per week will be obtained automatically through the study using heart rate data recorded by the wearable smartwatch. Specifically, the maximal heart rate will first be predicted using the following formula: maximal heart rate = 207 − (0.7 × age) [33].

**Table 1:** Data collected at each time point of the study.

	Baseline: day 0	Day 1-14	Daily from baseline	Thrice weekly (alternate days)	Weekly	End of the study (4 months +7 or −7 days)
Eligibility	✓					
Informed consent	✓					
Demographics	✓					
Height, weight, BMI, and waist circumference	✓^a^					✓
WHO^b^ performance status	✓					
Medical history or concomitant conditions and medications	✓					✓
Cancer type and stage	✓					
ED-5D-5L	✓^a^					✓
MFI^c^-10	✓^a^					✓
SATED^d^	✓^a^					✓
PACIC^e^	✓^a^					✓
PAM 13^f^	✓^a^					
Issue of smartwatch and Android device if required	✓^a^					
Food diary				✓		
Weight					✓	
Physical activity appointment including cardio-respiratory fitness and muscular strength		✓				
GPAQ^g^		✓				✓
Habitual physical activity assessment		✓				
Minutes of activity done by patient including strength and balance					✓	
Borg’s CR100 rating of perceived exertion scale					✓	
ESAS^h^			✓			✓
Continuous smartwatch data collection	✓^i^	✓	✓			
Usability questionnaire						✓
Adverse event assessment	✓^i^	✓	✓	✓	✓	✓
Collection of UKONS^j^	✓^i^	✓	✓	✓	✓	✓

^a^Conducted in person at baseline assessment visit.

^b^WHO: World Health Organization.

^c^MFI-10: Multidimensional Fatigue Inventory Short Form.

^d^SATED: Satisfaction, Alertness, Timing, Efficiency and Duration.

^e^PACIC: Patient Assessment of Chronic Illness Care.

^f^PAM-13: Patient Activation Measure–13.

^g^GPAQ: Global Physical Activity Questionnaire.

^h^ESAS: Edmonton Symptom Assessment System.

^i^To be collected from informed consent onward.

^j^UKONS: United Kingdom Oncology Nursing Society.

If a patient obtains a higher than predicted maximal heart rate during exercise, the actual maximal heart rate will replace the predicted maximal heart rate. The resting heart rate will then be recorded automatically by the watch. Heart rate zones will then be used to identify habitual physical activity intensity: very light (<30% heart rate reserve [HRR]), light (30%-39% HRR), moderate (40%-59% HRR), vigorous (60%-89% HRR), and near maximal to maximal (≥90% HRR) [34].

Energy expenditure during structured exercise will be estimated from the power output calculated from the mode of exercise, distance completed, and floors climbed. Other measurements such as body fat percentage, oxygen saturation, and blood pressure will be obtained using the standard features available within the smartwatch and smartphone.

The smartwatch will also prompt the participant to complete the ESAS questionnaire on a daily basis and to self-report the amount of habitual physical activity using the 4 abovementioned unvalidated questions on a weekly basis.

The SH app uses the Samsung Galaxy Watch to monitor sleep patterns, track sleep movements, and generate a sleep score. Additionally, the SH app provides daily goals and coaching programs based on sleep data to help improve sleep habits. Sleep analysis uses Samsung proprietary algorithms currently available through the public release of the SH app.

The SH app also allows the self-logging of the type and timing of meals and snacks. It is possible to set a daily target for caloric intake. After inputting details of the food type and quantity from a menu of common meals, the SH app provides a real-time view of the current calorie count for the day and a summary of the nutritional intake to determine if one is within the recommended levels. Moreover, it provides access to an overall dietary report over the span of a week.

### Contact From the Research Team

To ensure compliance with the trial, a telephone call by a member of the research team to all participants will be arranged at week 1 and then, if required, every 4 weeks until the end of the trial data collection period. The nature of these calls is to resolve any problems or concerns. The frequency of calls will be arranged and adapted by the research team members on an individual basis according to each participant’s needs and wishes. On the basis of prior experience with digital monitoring in patients with cancer [19], we believe that offering a regular opportunity to speak to someone who can help reassures participants, reduces anxiety, and in turn facilitates more accurate and consistent data collection.

### Concomitant Care Permitted During the Trial

During the study period, each participant will receive the indicated surveillance and routine care in accordance with their needs and clinical condition. Thus, any form of intervention for the underlying cancer or any intercurrent issue will be allowed (and recorded). Similarly, any type of diagnostic investigation, workup, or health care provider encounter will be permitted. To maximize participants’ safety, multiple measures have been put into place.

We will provide technical support and contact information for any inquiries, along with an escalation plan for clinical needs. This support includes online assistance, drop-in clinics, and remote technical support during office hours. Patients will be directed for any medical concerns to contact the Acute Oncology Hotline: 24/7 availability of the Acute Oncology Hotline support, to which participants have been accustomed during the on-treatment period in Alaw (oncology department in Ysbyty Gwynedd, Bangor, United Kingdom). The occurrence of any unplanned health care encounter (with general practitioner, district nurse, practitioner nurse, specialist nurse, dietician, physiotherapist, psychologist, occupational therapist, clinical pharmacist, or any hospital specialist) will be recorded with a weekly question through the GK app. Finally, members of the research team will be given access to a web-based portal, where they can log in and access patient data. Although data will not be checked with regard to patient clinical condition (the portal is designed to check only if data have been collected or not), for safety reasons, if when checking for data availability, the research team will notice any symptom with a severity score of ≥7 (on a 10-point scale) or any other potentially serious medical issue, the lead clinician or the consultant oncologist of the week (available 24/7) will be informed, and they will decide accordingly whether it is sensible to contact the participant to inquire about their complaint and select the most appropriate action.

### Primary Outcome

The main outcome of this trial is to assess the feasibility of mHealth monitoring in survivors of cancer not receiving toxic anticancer treatment. A priori criteria based on a traffic light system will be used to determine whether mHealth monitoring is unfeasible and future trials should be halted (red), whether changes to the study design or mHealth intervention are required before future trials are initiated (amber), or whether mHealth monitoring is considered feasible, and progress to future trials can occur without modification of the study design or intervention (green) [35]. The traffic light system will be applied at the end of the 16-week study period following collection of the last follow-up assessment from the last participant (on an intention-to-treat basis) to the following feasibility outcomes: eligibility (no set criteria but number of patients screened and eligible will be recorded); recruitment rate (green: n =>75, amber: n =≥50, and red: n=<50); acceptability of randomization and assessment procedure (no set criteria but the characteristics [and the proportion by group] of participants withdrawing post randomization will be captured); mHealth acceptability (green: ≥50/100, ≥50% of participants engage with the app at least once a week on at least 12 weeks out of the 16-week study, amber: ≥50/100, ≥50% of participants engage with the app at least once a week on at least 8 weeks out of the 16-week study, and red: <50/100, <50% of participants engage with the GK app at least once a week on at least 8 weeks out of the 16-week study); attrition rate (green: 20/100, <20% dropout rate, amber: ≤40/100, ≤40% dropout rate, and red: <40/100, >40% dropout rate); and missing data (green: <20/100, <20% of data missing, amber: ≤40/100, ≤40% of data missing, and red: >40/100, >40% of data missing). Engagement with the GK app is defined as logging on and completing at least 1 of the scheduled measures (ESAS, food intake, or habitual physical activity).

### Secondary Outcomes

**Figure 2:**
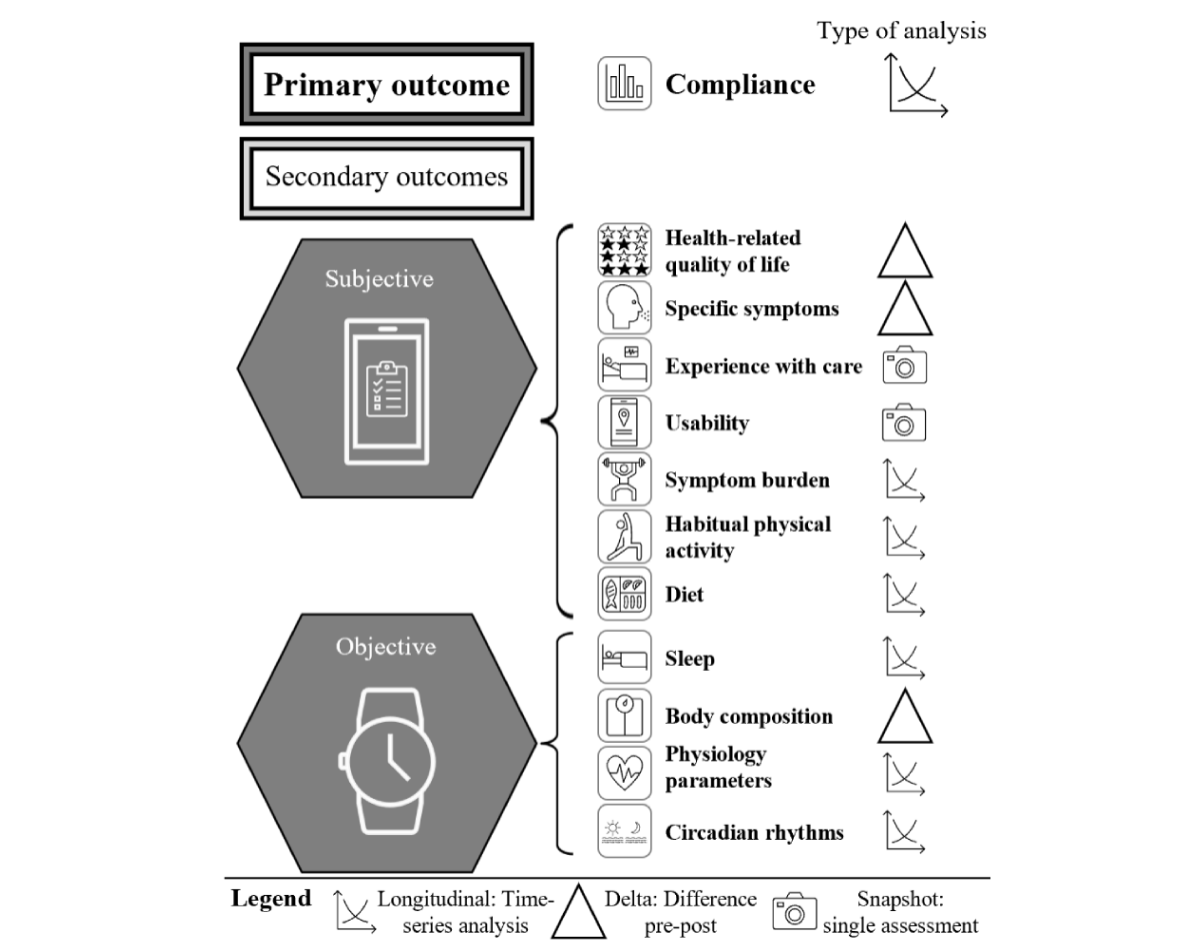
Summary of the pilot primary and secondary outcomes, which are categorized as subjective (from questionnaires) and objective (measured or from the smartwatch).

All other measures (as described in the *Study Procedures* section and in Figure 2) are secondary outcomes. They will be obtained to explore the direction and effect size of any changes as a groundwork for the development of future trials with meaningful end points, if appropriate. HRQoL will be further used in cost-effectiveness analyses. Within GK, cost-effectiveness is being conducted with the Monitoring and Assessment Framework for the European Innovation Partnership on Active and Healthy Ageing (MAFEIP) tool [36] to support evidence-based decision-making processes for all institutions and users in the health care sector. The main objective of the MAFEIP tool is to estimate the outcomes of a large variety of social and technological innovations by providing an early assessment of the likelihood that interventions will achieve their anticipated impact. In addition, MAFEIP also helps identify the drivers of a technology’s effectiveness or efficiency to guide further design, development, or evaluation. To conduct the cost-effectiveness assessment, all costs will be included in a comprehensive impact assessment (eg, hospitalizations, emergency department admissions, and procedures). The costs for treatment as usual will be collected from historical evidence to complement the comparison.

The patterns over 24 hours of accelerometry and heart rate measurements collected *continuously* (epochs defined through user settings ranging from hourly to continuously during workouts) through the smartwatch will be analyzed to retrieve relevant circadian (ie, of approximately 1 day) physiology parameters through cosinor, nonparametric, and hidden Markov model methods [37-40]. The circadian biorhythms parameters that will be retrieved integrate the endogenous body clock [41], physical activity, and sleep cycles [42]. Circadian rest-activity rhythm has shown relevance in oncology with regard to the well-being and overall survival of patients with cancer [43-45].

At 2 and 4 months, participants will also be asked to complete a usability questionnaire to obtain a System Usability Score [46] and a Net Promoter Score [47]. In addition, patients will be invited to attend a participant focus group. The focus group question guide will include two aspects: (1) experience of using the app and smartwatch during the previous 16 weeks and (2) a beta version of a future iteration of the app will be presented to the patients, and potential acceptability of the new features of this beta version will be explored.

### Exploratory Outcomes

The exploratory end points include the assessment of the precision (sensitivity and specificity) of AI-generated prediction of events based on the longitudinal patient-generated data sets. Specifically, events of interest will be severe symptoms requiring a call to the Acute Oncology Service (ranked amber and red on the United Kingdom Oncology Nursing Society triage tool [48]) or relapse (recurrence or progression) of cancer. Machine learning techniques applied to longitudinal datasets will be used, based on our previous experience [49].

### Data Management

Data in this study are multidimensional and will be collected via a range of sources including paper-based CRFs, paper-based and electronic questionnaires (completed via the GK app), and biometric data obtained from the smartwatches. All anonymized data collected by the NHS research team via paper-based methods will be transferred into a secure digital database, in a locked room, and on a password-protected desktop computer, whose access will be logged and monitored and limited to the research team members involved in the study.

Raw accelerations and heart rate data collected through the smartwatch will be periodically and seamlessly downloaded and sent to the GK app onto the paired smartphone. Afterward, once a day, these data will be automatically and securely uploaded onto the GK repository server through the internet in an anonymized fashion, where they will be kept for further time-series analyses, as previously described.

### Confidentiality and Data Protection Considerations

The sponsor (Betsi Cadwaladr University Health Board) has undertaken a full data protection impact assessment. To protect the confidentiality of participant data, paper-based CRFs and questionnaires will be linked to the participant via a trial identification number and stored in a separate location in the Investigator Site File containing the original consent forms. All paper-based documents will be stored in a locked cabinet in a restricted access office area. Participants will not be required to provide any personal information (such as name and date of birth) to register or use the GK app; instead, they will use the provided study account. The GK app will pull and anonymize data from the SH app, excluding contact details and other information that could be used to reidentify participants. The key linking the participant with the data set will only be held by the NHS research team and never be shared with other organizations.

### Trial Management and Risk Assessment

The sponsor has undertaken a risk assessment in line with the sponsor’s standard operating procedures, and the overall trial has been assessed as moderate risk, principally in relation to the multidimensional monitoring. The trial conduct and progress will be monitored by a trial management group including independent experts and patients to provide advice and support. A trial risk-adapted monitoring plan has been developed to check the research procedures; evaluate overall safety; and assess protocol compliance, data accuracy, and completeness.

### Statistical Considerations

The study’s primary outcome is feasibility, which, by design, is assessed by multiple outcomes (as described above). Arguably, the most important of these is patient engagement with the mHealth solution, and we have defined the solution as feasible if at least 50% (50/100) of the participants engage with the app at least once a week in 8 of the 16 study weeks. If we recruit 100 eligible participants, we will be able to estimate an engagement rate of 50% (n=50) with a 95% CI of +10% or −10%, whereby the width of the CI (in percentage) was calculated as follows:

1.96×√(p×(1−p)/n) **(1)**

where p is the percentage (50%) and n is the intended sample size (100). For this feasibility study, we considered this CI appropriate for estimating drop-off rates in future studies. Finally, we considered this sample size suitable for the preplanned exploratory subgroup analyses.

The main analysis of the primary outcome (compliance) will be performed on an intention-to-treat basis. Similarly, the secondary outcomes will be evaluated on an intention-to-treat basis. However, for the secondary outcomes, preplanned subgroup and sensitivity analyses will also be conducted. They will include (1) subgroups with at least 75% (75/100) of available patient-generated data (from the app and the smartwatch combined) to exclude unengaged participants and focus on the most committed participants, (2) subgroups defined by the performance status and presence or absence of evidence of neoplastic disease, and (3) subgroups defined by sex and median age. For the primary outcome, no form of inference will be performed on the missing data, as they provide the main outcome of the study. For secondary analyses, neither mean imputation nor the last observation carried forward will be used [50]. However, based on the proportion of missing data, exploratory analyses including low-rank approximation–based imputation [51] could be performed to ensure that the observed effect sizes remain of the same order of magnitude with this approach as well.

## Results

As of January 8, 2024, we are performing the last preparatory steps before enrollment, such as finalizing letters for GPs and internal communication to the oncology department staff about the study, its finalities, and referral criteria.

We anticipate enrolling the 100th patient by the end of June 2024. Thus, the last patient’s last visit is expected in late November 2024. We foresee an additional month to complete the data collection and solve the pending queries. Data analyses will then be performed once all data have been collected, and the database will be frozen. Primary and secondary outcomes will be prioritized over the exploratory and sensitivity analyses. The overall evaluation of the study will be presented as part of the results of the GK large-scale pilot to the EU Commission and via the project web portal.

The main outcomes will be submitted for publication to an appropriate peer-reviewed scientific journal and, if opportune, to a scientific meeting in the domains of oncology, digital health, behavioral medicine, and sport and exercise science. Additional reports of specific findings elaborated more in depth will be considered for submission for scientific publication or presentation, according to the pertinence of the results obtained and their clinical and scientific relevance.

## Discussion

### Study Goals and Expected Findings

This study aims to determine the feasibility of self-monitoring of symptoms, biometrics, and behavior using an mHealth solution. If feasible, the efficacy of the mHealth solution will be addressed in subsequent studies. Ultimately, this study has two synergic objectives: (1) The first concerns the improvement of the quality of life and expectancy of independent active life. (2) The second concerns the cost-benefit to health care systems facing an increasing number of older adults and an increase in life expectancy, which is not compensated by a comparable increase in budget and staff. Regarding the first concern, in the case of patients with chronic conditions, this expectation should be considered in the context of the detrimental effects of long-term treatments and stress and anxiety related to the monitoring period. Regarding the second concern, the ultimate goal of the mHealth solution is to lower the likelihood of hospitalization, the worsening of patients' conditions, and the resulting complexity of life-long treatments. In this regard, the mHealth solution we propose to evaluate here for acceptance and compliance aims to ultimately lower the risks of worsening and adverse events via a combination of (1) early prediction leading to early intervention, and (2) prevention through behavioral intervention focused on the wide-spread adoption of good healthy lifestyle practices.

Technology is a key enabler in achieving these synergistic objectives. On the one hand, devices and apps lower the cost of monitoring while improving the variety, granularity, and frequency of data. For instance, apps exploit patients’ hardware, freeing up time during appointments to be used to discuss patients’ well-being. In the context of cancer survivorship and unmet care needs, the use of digital tools to try to overcome some of the barriers to the adoption of a healthy diet, sleep, and exercise behaviors is particularly relevant from the clinical, scientific, and health economics standpoints.

Thus, this observational pilot study has been tailored to provide feasibility evidence in view of future interventional studies aimed at specifically addressing some of the unmet needs of survivors of cancer. The personalized promotion of healthy habits, including diet, sleep, and physical activity, as pillars of supportive care in survivors of cancer combined with the monitoring of signs and symptoms is expected to improve (or at least sustain) HRQoL. Moreover, given its relatively inexpensive implementation and the potential for benefits, we expect such an intervention to be cost-effective.

With this objective in mind, we have selected multiple secondary end points, all relevant for gaining insight into (1) the effect size of the clinical impact of the mHealth solution on symptom burden, HRQoL, and perceived care; (2) the temporal patterns of physical activity, diet, and sleep in survivors of cancer; and (3) the usability of such digital solutions. In our opinion, collecting these dense, multidimensional, longitudinal patient-generated data is paramount for the development of new forms of tailored behavioral interventions. Indeed, initial evidence in oncology is being produced, testing integrative behavioral approaches to improve cancer-associated and treatment-induced symptoms [5,52-58].

Along with these observational outcomes, patient safety remains paramount in our pilot and in all future interventions [59,60]. Therefore, besides the patient-prompted 24/7 acute oncology helpline, we have also put in place a safety net by which the clinical research team will have access to the web-based dashboard summarizing all the ESAS scores and the smartwatch objective data, with the possibility of escalation to the leading oncologist in case of severe symptoms or profoundly abnormal vital data. Moreover, as part of the exploratory analyses, we plan to use AI to investigate whether adverse events (unplanned health care provider encounter, emergency admission, or severe symptoms) could be reliably predicted between 3 and 5 days in advance from the temporal patterns of the collected objective and subjective patient-generated data [61-64]. Finally, we plan to use the same AI-enabled predictive approach to explore the accuracy of predicting cancer relapse at least 3 weeks before formal clinical suspicion. However, we expect relatively few such events during the study period.

### Strengths and Limitations

The main limitations of this study include the choice of a very heterogeneous population of survivors of cancer, at very different stages of the clinical trajectory of their disease, as well as marked diversity in terms of prognosis. This was a deliberate choice for our first pilot, influenced by recruitment time constraints exacerbated by the disruption in research delivery caused by the COVID-19 pandemic. Associated with this is the monocentric nature of the study, a limitation in terms of potential generalizability but a compelled choice for an initial testing of the digital solution in a pilot feasibility trial.

We also acknowledge that the behavioral medicine approach deployed in this study has been derived mainly from published evidence [65,66] and minimally from in-house expertise. Thus, we have consciously selected to simplify our behavioral change approach, for example, avoiding ecological momentary assessments [67] or interpersonal social rhythm interventions [68].

We also accept that technology faces some barriers to adoption. From digital literacy to internet access, a large segment of the older adult population does not have or does not wish to use digital technology. Although this is likely a temporary challenge, characteristic of a demographic transition, it provides the need or opportunity to design noninvasive solutions that blend within our activities and habits. In the case of patients with chronic conditions, this type of design can be seen as part of the effort in mitigating the long-term effects of having to manage their conditions and treatments on their mental health and well-being.

Despite these limitations, we believe that this pilot study is both timely and relevant. Digital monitoring in oncology is becoming increasingly widespread and often integrates both patient-reported outcomes and data from a wearable biosensor [69-73]. We focus here on survivors of cancer after treatment, a population in which gaps in research have been identified [73]. Integrating in the future, an interventional approach of tailored coaching for behavioral change alongside the longitudinal multidimensional digital telemonitoring, we trust this pilot can provide unique and novel insight into the clinical relevance of predictive, preventive, personalized and participatory (P4) medicine in survivors of cancer [74]. Thus, we anticipate that if feasibility is demonstrated with this pilot study, we will test a novel form of AI-enabled digital behavioral intervention in a future dedicated randomized study. Such a future intervention could include (1) an initial dietary, sleep, and physical activity consultation; (2) behavioral monitoring recorded via a smartphone app and wearable smartwatch; (3) monitoring of symptoms and health medical records; (4) integration of data to allow delivery of AI-generated, personalized digital coaching on nutritional and physical activity behaviors; and (5) development of risk prediction algorithms to allow early identification and management of cancer-related symptoms, hospitalization events, and cancer relapse.

### Conclusions

As part of the GK large-scale pilot, this study (its design, anonymized data, results, and technologies) will be made available to other selected health care organizations. These organizations, such as regional health care agencies and hospitals, will assess and evaluate the proposed approach with the aim of transferring the lessons learned from the study and, ideally, readapting and adopting this approach to their context, resources, and constraints. These activities are part of a specific *twinning* work strand that is open to both GK Reference Pilot Sites and third-party health care organizations interested in practices exchange and in being early adopters of new AI solutions.

This study is one of the many being developed within the scope of the GK project, all of which share the underlying aim of improving the quality of life of the aging population and people living with chronic conditions. However, this is 1 of the only 2 GK studies focused on cancer, with a study in Cyprus focusing on palliative care for the end of life [13]. As such, this is the only pilot in the GK large-scale pilot that addresses the challenges of long-term monitoring of cancer symptoms. Although its uniqueness is limited only to the GK projects, this study provides essential input to understand how a fairly common technological setting (a mobile app and wearable) can be adapted to this scenario and be potentially used at scale for lifelong monitoring of survivors of cancer. This type of study generates an unprecedented quantity of multidimensional and longitudinal datapoints. It provides an opportunity but also a new set of risks. Indeed, part of the GK project activities concern the legal, policy, and practical arrangements necessary to move, share, combine, and reuse patient data. This is a significant challenge that can be addressed through this type of study, which is also aimed at identifying hidden barriers. Furthermore, this new setting presents both a challenge and an opportunity to redefine the relationship between patients and caregivers. It raises questions about the optimal use of data to promote dialogue and enhance understanding of the recovery journey after discharge, such as from a cancer support service.

## Abbreviations

AI: artificial intelligence
CRF: case report form
ESAS: Edmonton Symptom Assessment System
EU: European Union
GK: GATEKEEPER
HRQoL: health-related quality of life
HRR: heart rate reserve
MAFEIP: Monitoring and Assessment Framework for the European Innovation Partnership on Active and Healthy Ageing
mHealth: mobile health
NHS: National Health Services
SATED: Satisfaction, Alertness, Timing, Efficiency and Duration
SH: Samsung Health

## Data Availability

The data sets generated and analyzed during this study will be made available in the Open Research Online repository of the Open University.
